# Eye Exercises Enhance Accuracy and Letter Recognition, but Not Reaction Time, in a Modified Rapid Serial Visual Presentation Task

**DOI:** 10.1371/journal.pone.0059244

**Published:** 2013-03-19

**Authors:** Paula Di Noto, Sorin Uta, Joseph F. X. DeSouza

**Affiliations:** 1 Centre for Vision Research, York University, Toronto, Canada; 2 Neuroscience Graduate Diploma Program, York University, Toronto, Canada; 3 Department of Psychology, York University, Toronto, Canada; 4 Department of Biology, York University, Toronto, Canada; 5 Canadian Action and Perception Network (CAPnet), Toronto, Canada; CNRS - Université Claude Bernard Lyon 1, France

## Abstract

Eye exercises have been prescribed to resolve a multitude of eye-related problems. However, studies on the efficacy of eye exercises are lacking, mainly due to the absence of simple assessment tools in the clinic. Because similar regions of the brain are responsible for eye movements and visual attention, we used a modified rapid serial visual presentation (RSVP) to assess any measurable effect of short-term eye exercise in improvements within these domains. In the present study, twenty subjects were equally divided into control and experimental groups, each of which performed a pre-training RSVP assessment where target letters, to which subjects were asked to respond to by pressing a spacebar, were serially and rapidly presented. Response time to target letters, accuracy of correctly responding to target letters, and correct identification of target letters in each of 12 sessions was measured. The experimental group then performed active eye exercises, while the control group performed a task that minimized eye movements for 18.5 minutes. A final post-training RSVP assessment was performed by both groups and response time, accuracy, and letter identification were compared between and within subject groups both pre- and post-training. Subjects who performed eye exercises were more accurate in responding to target letters separated by one distractor and in letter identification in the post-training RSVP assessment, while latency of responses were unchanged between and within groups. This suggests that eye exercises may prove useful in enhancing cognitive performance on tasks related to attention and memory over a very brief course of training, and RSVP may be a useful measure of this efficacy. Further research is needed on eye exercises to determine whether they are an effective treatment for patients with cognitive and eye-related disorders.

## Introduction

Eyesight is the most evolutionarily advantageous sense for human beings. It allows for simultaneous, rapid, and efficient processing of information from the environment. This information is subsequently used to facilitate numerous cognitive functions, such as perceiving possible hazards and observation-based learning. Based on the immense reliance we have upon our vision, it is no wonder that many clinical techniques have been developed to assess and treat a multitude of eye and eye-related problems. Eye exercises are often prescribed in vision therapy to resolve issues relating to vergence, ocular motility disorders, accommodative dysfunction, amblyopia, learning disabilities, dyslexia, asthenopia, myopia, motion sickness, sports performance, stereopsis, visual field defects, and visual acuity [1]. Eye exercises are also practiced to enhance sports performance and used during yoga to promote general well-being [2]. However, few studies exist on evaluating the outcomes and efficacy of this type of therapy [1], [3]–[9]. One such study found a measurable effect of eye exercise in patients with convergence problems [10]. Additional research suggests eye exercise facilitates improvement in stereoscopic skills and visual field remnants after brain damage [11]; and there has been evidence to link visual attention and visual working memory (for review see [12]). Aside from these areas, there exists no other research implicating eye exercise as an effective treatment for other types of visual or cognitive deficits. Despite this lack of empirical peer reviewed research, eye exercise remains a popular technique/therapy as demonstrated by a Google search of “eye exercise” conducted in April 2009 which yielded 13,400,000 results, and 244,000,000 results when searched again on April 3^rd^, 2012.

Perhaps the most popular type of vision therapy is the ‘See Clearly’ technique established by ophthalmologist William Horatio Bates [2]. His book, *The Cure of Imperfect Sight by Treatment without Glasses* [13], highlights a wide assortment of possible therapeutic eye exercises, one of which requires patients to shift their fixation between two targets repeatedly without staring. This is intended to relax the eye and correct errors in refraction leading to improved vision. Essentially, Bates’ eye exercise technique employs rapid saccadic eye movements in an attempt to improve visual *acuity*, but there is no discussion in his work on how saccades affect visual *attention*. Through a review of historical literature and our present investigation, we intend to illuminate the mystery of eye movement training and its relation to visual attention.

Evidence in the field of vision research has suggested the frontal eye fields (FEF), an oculomotor region of the premotor cortex, is of paramount importance during voluntary saccade production in humans and non-human primates [14]–[16]. Pro-saccades are small, jerky eye movements towards an object or cue. Anti-saccades are, by definition, gaze shifts in the opposite direction of a presented stimulus cue [17]–[20]. Munoz and Everling [21] reviewed single neuron activity in the FEF and found it to be responsible for voluntary control of anti-saccade eye movements, which are guided by instructions and attentional control. Further to this, inactivation of the FEF via chemical injection has resulted in an abolition of voluntary saccades to the contralateral visual field [22]. The FEF is also shown to be involved in visual attention tasks [16], [23], [24]. Thus, we can reasonably suspect that repeated saccade performance will have a measurable effect on the visual attention network depending on the amplitude and frequency of saccades. Since the synapses of neurons are dynamic and adaptive [25], [26], it is theoretically possible to improve performance on a visual attention task by strengthening the connections of neurons through saccadic eye exercise over a short period of time, such as a few hours or even a few minutes.

A recent study by Dyckman and McDowell [27] involved training participants on one of three eye movement tasks (anti-saccade, pro-saccade or fixation). Participants trained on the pro-saccade task made more errors on subsequent anti-saccade testing, while subjects trained on the fixation task showed no change in errors during the pro- and anti-saccade testing sessions. Subjects that underwent anti-saccade training had decreased errors while maintaining the same speed. These findings suggest that deliberate practice of eye movements can alter anti-saccade performance on later testing, and the enhancement of accuracy demonstrated in the anti-saccade training group was not a trade off for speed. Furthermore, an assertion was made that the direction of the practice effect depends on the type of training task one engages in such that the subjects’ performance would improve on the task they had been trained on. While the effects of saccade practice have been shown to alter one’s performance on anti-saccade tests, much remains unclear regarding its effects on visual attention performance and the putative involvement of the FEF region in relation to saccade practice effects and visual attention. What these studies do underscore, however, is the highly plastic nature of the brain and visual system, which can adjust to different requirements with sufficient training [25], [28] (for review see [29]).

To the best of our knowledge, there exists no study that assesses changes in visual attention and memory following vision training with eye exercises. The majority of studies assessing the progression of vision-related problems utilize the subjective College of Optometrists in Vision Development (COVD) quality of life checklist [30]. The 30-item questionnaire requires patients to input the frequency of personally experienced issues related to Physical-Occupational (mobility), Social Integration (personal relationships), Somatic Sensation (physical symptoms) and Psychological (overall life satisfaction) domains. Studies on patients that undergo this therapy while being evaluated with the COVD checklist show a decrease in subjective symptoms and an improved overall quality of life [31]. However, there is an absence of any evaluation of the quantitative behavioural or putative neural changes in the vision-related problems that subjects present with.

Because of the lack of assessed improvements in vision, attention, and working memory following therapeutic intervention, the present study was designed in an attempt to investigate whether eye exercises as visual training influence performance on a visual attention task. Dyckman and McDowell [27] indicated their observed practice effect can be quantified in as little as 3 days. However, the present study will attempt to demonstrate improvements in visual attention after only 18.5 minutes of eye exercises as measured by rapid serial visual presentation (RSVP). Joseph, Chun and Nakayama [32] demonstrated that RSVP is a sensitive tool capable of measuring visual attention through letter identification. This tool is especially fitting for the present investigation, which provides a limited but sufficient amount of visual training in an experimental paradigm that can be easily replicated and performed in under 20 minutes. Our paradigm will divide subjects into two groups based on their training condition: an active eye exercise group and a control group that will perform minimal eye movements. Both groups will perform RSVP twice (prior to and following their respective training conditions), which ensures that practice effects related to RSVP exposure can be eliminated and any differences in RSVP performance can then be attributed to the effects of eye exercises on visual training. Following the assumptions of Dyckman and McDowell [27], we hypothesize that participants who undergo eye exercise will respond to RSVP with greater accuracy in a second post-training evaluation while maintaining similar response times. Similarly, we do not expect the control group to exhibit any changes in speed or accuracy during the subsequent RSVP task. If a measurable difference in visual attention is indeed detected in the post-training RSVP task, we can lend further evidence to the efficacy of eye exercises as a useful method of enhancing visual and cognitive control.

## Methods

### Subjects

**Figure 1:**
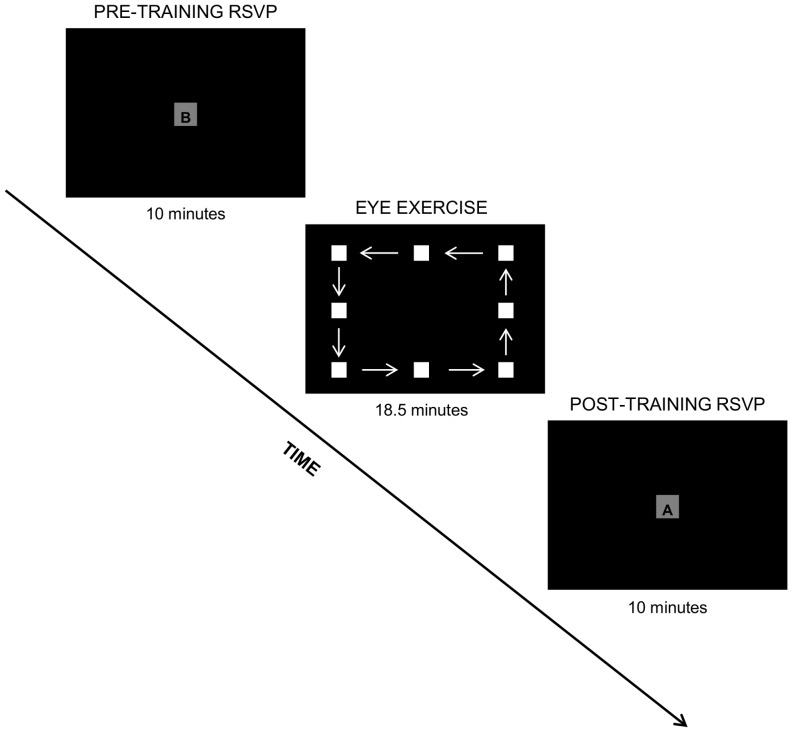
Experimental design for the eye exercise group. Following a 10 minute RSVP task, subjects in the eye exercise group were instructed to follow a moving box on the screen, effectively performing numerous saccadic eye movements, before being assessed in a second RSVP task.

**Figure 2:**
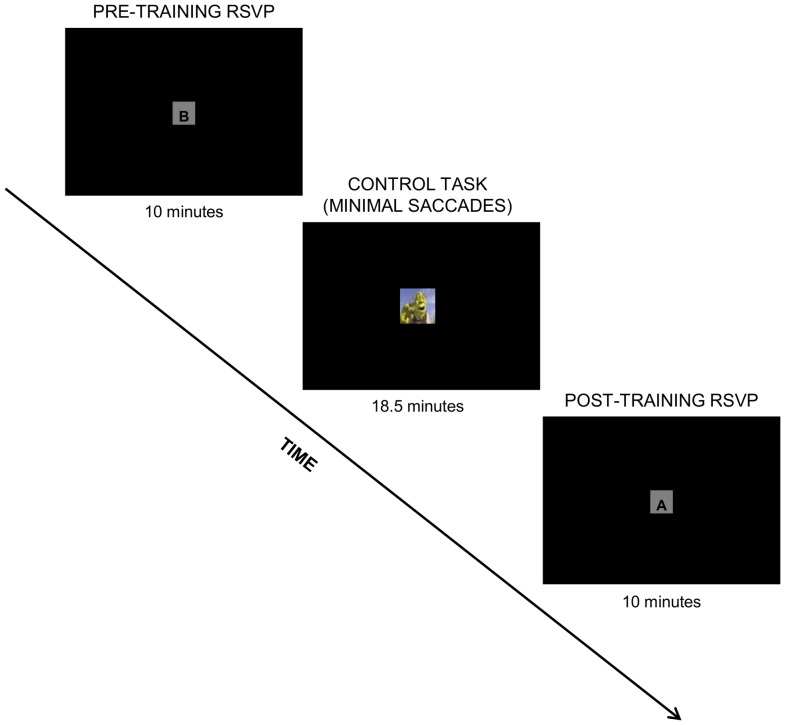
Experimental design for the control group. Following a 10 minute RSVP task, subjects were asked to fixate on a movie clip presented in a very small box on the screen, effectively performing a very limited amount of eye movements, before being assessed in a second RSVP task.

Twenty participants (10 female, mean age = 26.0, SD = 11.2) volunteered for the present study. All participants were free of memory problems and uncorrected vision problems. Participants were randomly assigned to one of two age- and sex-matched groups: the experimental group (5 female, mean age = 27.3, SD = 11.2) or the control group (5 female, mean age = 24.8, SD = 11.7). These groups were exposed to two different training conditions, which are illustrated in Figure 1 for the eye exercise group and in Figure 2 for the control group.

### Ethics Statement

The present study was approved by the York University Human Subjects Review Board and conducted in accordance with the Declaration of Helsinki. All participants provided signed consent prior to taking part in the study, and were free to withdraw at any time with no consequences.

### Rapid Serial Visual Presentation (RSVP)

**Figure 3:**
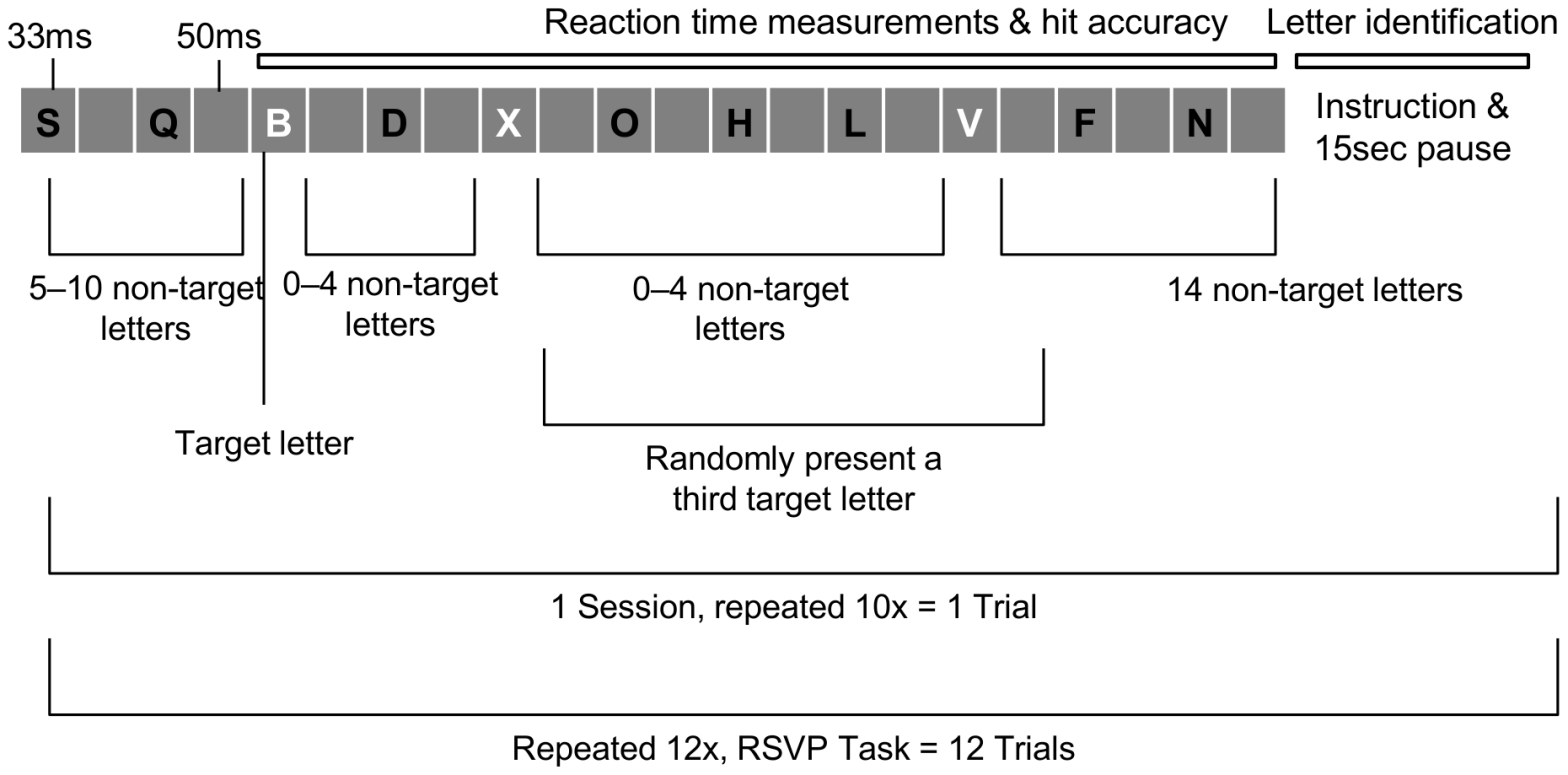
Experimental paradigm for the modified RSVP task. All letters were presented at central fixation, with target letters appearing in white and non-target letters appearing in black font.

For both eye exercise and control groups, participants were assessed using RSVP before and after their respective training conditions. In RSVP, all 26 letters of the English alphabet are randomly presented in either black (RGB: 0, 0, 0) or white (RGB: 255, 255, 255) within a 30×30 pixel grey box (RGB: 128, 128, 128) at the center of the screen. Black letters are non-target letters while white letters represent the target to which subjects responded by button press on a computer keyboard. Each letter is presented for 33 ms followed by a 50 ms blank box. A trial starts with the presentation of 5 to 10 non-target letters followed by a target letter. Following presentation of the first target letter, another 0 to 4 non-target letters are shown before the next target letter. Either 2 or 3 target letters are shown per session before concluding with 14 non-target letters. This sequence is repeated a total of 10 times to constitute one trial. Our subjects were exposed to 12 trials in total. Our RSVP task replicates the one used by Joseph and colleagues [32], except our paradigm includes second and third additional target letters that are randomly presented in the trials within each session. This was meant to ensure that each session randomly presented 2 or 3 target letters and participants were unable to predict how many times they were required to respond. The total experiment consists of 12 trials, each consisting of 10 sessions (1 session = 5−10 non target, target, [0–4 non target, target]×1 or 2 randomized, 14 non target) (Figure 3). Total time for the entire experiment of the modified RSVP task is approximately 10 minutes. We were interested in measuring three aspects of subjects’ performance in the RSVP task: reaction time to responding to target letters, accuracy in responding to target letters, and accuracy in identifying the final target letter in every tenth session.

### Letter Identification

Expanding upon the RSVP task presented by Joseph et al. [32] we incorporated an instruction to identify the target letters observed during every tenth session. Participants were prompted by an instructional screen to indicate the letters presented, and were given extra time (15 sec) to indicate the letters using the computer keyboard. The purpose of this evaluation was to determine whether eye exercise facilitated improvements in visual working memory as measured by letter identification, and specifically, with the number of correct responses. Overall, letter identification was required 12 times, once after the final session of every trial (Figure 3).

### Eye Exercise Task

The experimental condition had ten participants perform an active eye exercise task, which involved following a white square on a black background through a series of paths described as follows. The participants were expected to make saccadic eye movements while following the square through a course of 5 horizontal figure eights, 5 large rectangles outlining the edge of the screen, 5 vertical figure eights and 5 small rectangular paths. This cycle was repeated 4 times. The square would move to a new position every 150 ms. The duration of the eye exercises was 18.5 minutes. Overall, following the square would have resulted in the performance of 7,740 saccadic eye movements, thus maximizing the amount of eye movements performed. The eyes of the subjects were observed by the experimenter during the entire task to ensure that subjects were actively participating in the task. All subjects complied with the instructions and were included in the analysis.

### Control Task

The control group had a training condition that intended to minimize the amount of eye movements, or exercise, that the participants engaged in. As such, the control task involved participants’ passive observation of an 18.5-minute clip of a movie (Shrek 3) without sound. This clip was displayed within a 50×50 pixel sized box surrounded by a white screen. The dimensions were purposefully small to minimize saccadic eye movements. Any changes found in the control group were expected to be due to effects of the RSVP task and thus can be used to factor out the practice effects from the experimental group. The eyes of the subjects were observed by the experimenter to ensure that the subjects were actively participating in the task.

## Results

### Reaction Time

We compared reaction time to responding to target letters in the pre-training versus post-training RSVP task with a repeated measures ANOVA and found that neither group deviated from their pre-training reaction time (training and test condition interaction: F(1,17) = 2.334, P = 0.145). One participant from the control group was excluded from analysis due to a malfunctioning button press and a loss of registered data. Paired sample *t* tests reveal that average reaction time for the eye exercise group did not differ from the control group in either the pre-training (*t*(8) = 0.429, P>0.1) or post-training RSVP task (*t*(8) = 0.145, P>0.1).

### Response Accuracy

Response accuracy was analyzed with a 2×2×5 repeated measures ANOVA (Table 1). Only the main effect of distractor was highly significant (F(4,72) = 113.72, P<0.001, η_p_^2^ = 0.863), with expected increases in response accuracy as the number of distractors between target letters increased from zero to four. The distractor*training interaction was approaching significance (F(4,72) = 2.175, P = 0.080) but had a large effect size (η_p_^2^ = 0.108), suggesting an influence on response accuracy by practice effects or training effects, presumably from the eye exercise condition. This final presumption is strengthened by the significant distractor*condition interaction (F(4,72) = 7.35, P<0.000), with the eye exercise group outperforming the control group at two (P = .022) and four (P = .030) distractors (both P values adjusted with a Bonferroni correction). Although non-significant, the training*condition interaction had a medium effect size (F(1,18) = 1.179, P = .292, η_p_^2^ = .061), and the three-way interaction between condition*training*group also demonstrated a small effect size (F(4,72) = .363, P = .834, η_p_^2^ = 0.020).

**Table 1:** Response accuracy during RSVP task.

	F (df)	P	Effect size (η_p_^2^)
Distractor	113.724 (4,72)	.000***	.863
Training	0.663 (1,18)	.426	.036†
Distractor×Training	2.175 (4,72)	.080	.108†††
Distractor×Condition	7.350 (4,72)	.000***	.290
Training×Condition	1.179 (1,18)	.292	.061††
Distractor×Training×Condition	0.363 (4,72)	.834	.020†

*** Significant at P<.001,

† Small effect size,

†† Medium effect size,

††† Large effect size.

**Figure 4:**
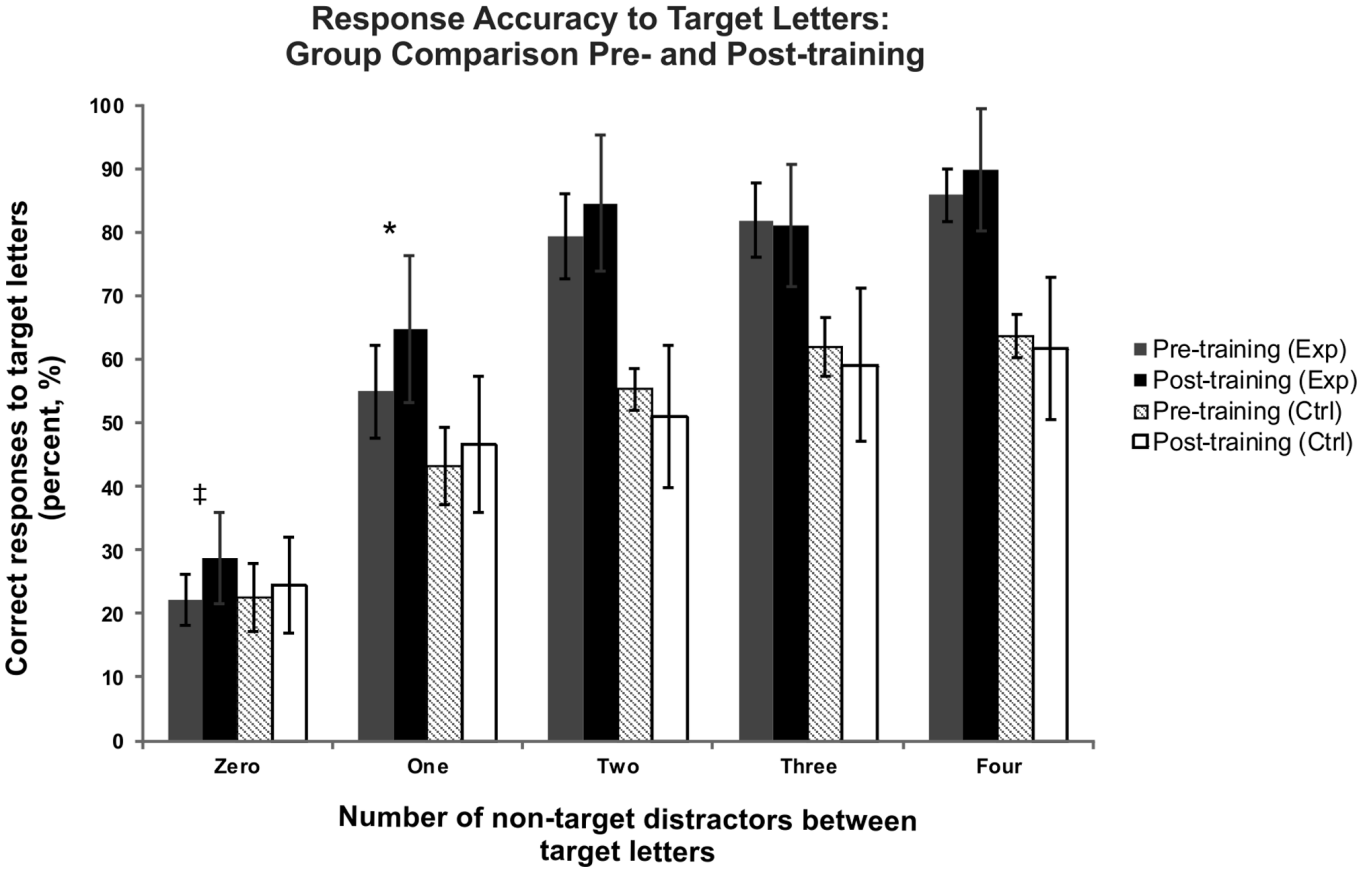
Comparing pre- and post-training response accuracy to target letters between groups. Subjects who were trained with eye exercise (grey and black bars) showed significant enhancement in responding to target letters separated by one distractor (‘One’ condition) following eye exercise training, and were approaching significance when responding to two sequentially presented target letters (‘Zero’ condition). This enhancement was not shown in the control group (cross-hatched and white bars), and there were no changes in either group’s accuracy of detecting targets separated by more than one distractor. *−P = 0.05, ^‡^−P = 0.079.

Despite the lack of statistical significance of the interactions implicated in eye exercise training, graphical examination of the data (Figure 4) together with the overlap of significant effects and noteworthy effect sizes prompted us to examine the three-way interaction further, as it is the most informative with respect to answering our main research question: does eye exercise improve performance on cognitive tasks of visual attention and memory? Pairwise comparisons adjusted with a Bonferroni correction show a significant improvement in response accuracy for the eye exercise group only when one distractor is presented between target letters (P = 0.050) and was approaching significance when target letters were presented sequentially (P = 0.079, black and grey bars in Figure 4). The control group failed to deviate from pre-training performance regardless of the number of distractors presented between target letters (P>.05). To clarify any inherent differences in response accuracy between groups pre-training, we conducted *post hoc* one-way ANOVAs for each level of distractor, which revealed subjects in the eye exercise group were better at responding to target letters separated by four distractors (F(1,19) = 4.55, P<0.05), but groups did not differ in pre-training accuracy in the remaining four distractor conditions (P>0.1). A composite performance measure comparing correct responses to false alarms (FAs, i.e., pressing spacebar in response to non-target letters) reveal a minimal influence of FAs on response accuracy (d′ = 4.12 pre-training for both groups, d′ = 3.84 post-training for both groups).

### Letter Identification

**Figure 5:**
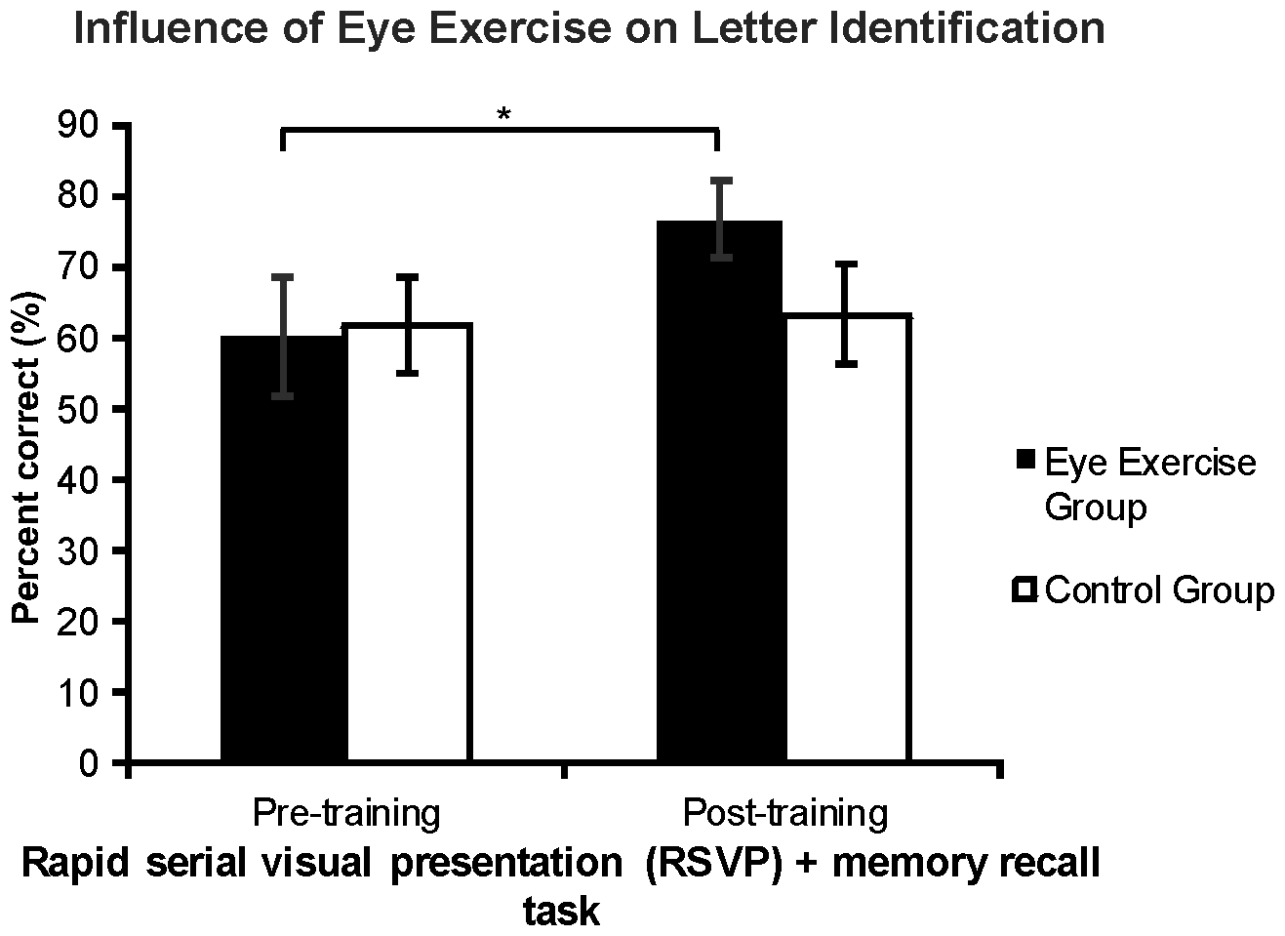
The influence of eye exercises on letter identification. Subjects who were trained with eye exercises showed a significant increase in the letter identification task following the post-training RSVP task compared to letter identification performed after the pre-training RSVP task. *−P<0.05.

Subjects in both the eye exercise group and control group were assessed on their ability to correctly identify the final target letter presented in each of the twelve trials per RSVP session. One participant from the experimental group was excluded from analysis due to a malfunctioning button press and lack of registered data. A repeated measures ANOVA revealed a significant increase in letter identification accuracy across both groups (F(1,17) = 5.138, P = .037), but with an interaction effect of training and condition only approaching significance (F(1,17) = 3.598, P = .075). When exploring the nature of this interaction further, pairwise comparisons reveal that only subjects trained with active eye exercises demonstrated significant improvements in letter identification (P = .011), while the control group failed to deviate from their pre-training performance (P = .791, Figure 5). All subjects performed well above the chance rate for guessing the correct letter throughout the experiment (i.e., 1/26 = 3.85%). and groups did not differ in pre-training letter identification accuracy (*t*(8) = −.438, P>0.1). Sensitivity analyses reveal that only the experimental group significantly reduced the relative number of errors in their performance (d′ = −0.04 pre-training, d′ = 0.77 post-training, paired-samples *t*(8) = −2.92, P<0.01), while the control group sensitivity measure did not change significantly (d′ = 0.07 pre-training, d′ = −0.09 post-training, paired-samples *t*(9) = 0.738, P>0.1). Errors were classified as incorrect letters reported (despite a correct response to target letters), no response and no reported target letters, too many letters identified both with and without responses to each target letter, no letters identified despite correct responses, no response and incorrect letters identified, and no response but correct letter identification.

## Discussion

The purpose of the present study was to determine whether eye exercises alter performance on a visual attention and memory task, which would be suggestive of vision therapy as a useful tool in treating similar and related cognitive and eye-related problems such as amblyopia, myopia, learning disabilities, motion sickness, stereopsis, visual field defects, and visual acuity. Previous studies have investigated whether vision therapy is an effective treatment for eye-related problems [30]; however, these studies did not evaluate any enhancements of cognitive performance. To the best of our knowledge, no direct quantitative measure of improvement on accuracy of eye movements, or in the treatment of impaired cognition, exists following a session of eye exercise. To conduct such an evaluation, we chose to use a simple RSVP task modified from the one used by Joseph et al. (1997). By using a task that demands very high attentional resources at central fixation, we can conclude that any significant improvements in performance can be attributed to enhancement as a result of eye exercise. These enhancements may be mediated by short-term changes in neural activity through eye exercise-induced priming and/or mechanistic short-term plasticity of frontal and parietal regions and superior colliculus, which are responsible for visual attention, preparatory motor signaling of the visuomotor system, and working memory [12], [15], [16], [20]–[22], [24], [33]–[35].

Neural plasticity is defined as a change in the activity and connections among populations of neurons as a result of experience-based modification in behavior. Importantly, short-term plasticity that occurs over a very brief time span, such as minutes or hours, involves the control and regulation of dynamic activity [26]. Although plasticity requires excitatory neural activation in order to occur, the former induces changes in *patterns* of neuronal activity in addition to improved behavioral performance, both of which are maintained long-term with continued practice [26], [27]. Although we did not measure changes in cortical activity in our purported neural network, previous work has correlated enhanced oculomotor performance with short-term neuronal plasticity [26]. Based on the observed behavioral improvements in response accuracy and letter identification, we can infer that eye exercises successfully primed this network by way of excitatory neural activity which with continued practice could potentially lead to short-term plasticity of the oculomotor circuit. Thus, a brief course of eye exercises may facilitate improvements in cognitive task performance through short-term changes in cortical activity, and could prove to be an effective short-term treatment for other cognitive and eye-related disorders.

There were at least three ways in which a subject could show an improvement in visual attention on the RSVP assessment: (a) faster reaction time when responding to a target letter, (b) overall increased accuracy in responding to target letters, and (c) improved accuracy of target letter identification.

In the present study, reaction times were not significantly different in the post-training session compared to the pre-training for either group. The lack of a difference between the two groups suggests that any significance found within the subjects would have been purely due to practice effects associated with RSVP experience. This conclusion is in line with the findings of Dyckman and McDowell [27], as subjects in their experiment did not experience changes in reaction time. Recently, it has been suggested that reaction time improves on a procedural visuo-motor task with the allowance of sleep following training [36]. Thus, future studies may need to incorporate a more dispersed regime of eye exercise before any significant improvement on reaction time can be seen. Furthermore, studies have also shown that introducing physical activity to a visual response task can improve reaction time [37]. An improvement was shown not only on tasks requiring a motor response (button press), but also on cognitive processes requiring inhibition of preplanned motor actions (suppressing response to an inhibitory cue). The effects of exercise were observed during, immediately after and at least 52 minutes after strenuous activity on an exercise bike. Thus, future studies may need to incorporate an element of physical activity to further examine its interaction with visual attention.

There were no changes to overall accuracy in response to target letters for the control group. However, following eye exercise, there was a significant increase in accuracy only when responding to two target letters separated by one non-target letter (P = .050), and was approaching significance when presented in sequence with no distractors between them (P = 0.079, Figure 4). Additionally, all main and interaction effects analyses including the training factor demonstrated at the very least small but notable effect sizes (Table 1). Although we hypothesized that subjects trained with eye exercises would show greater accuracy in responding to target letters than controls, we did not anticipate that the improvements would occur at only the first two levels of our five distractor conditions. We believe that the enhancement of detecting visual cues following a relatively brief (18.5 minutes) period of eye exercise during the two quickest serial presentations of target letters (presented between 50–133 ms apart) perhaps reflects the strongest evidence for enhancement of visual attention following eye exercises. That subjects trained with eye exercises were able to significantly improve their performance in one of the two most rapid levels of the RSVP task suggests a high level of reactivity and adaptability of the visuomotor and attentional systems, connected by a common neural network and putatively primed by eye exercises. That the experimental and control groups did not differ at higher levels of the distractor condition could reflect ceiling effects for reaction times to target letters presented with two or more distractors between them (i.e., at least 216 ms between presentation of two target letters). This finding is congruent with Dyckman and McDowell’s [27] findings, which demonstrate that response accuracy improved with eye exercise. While our exercise task involved only pro-saccadic eye movements to train for responding to a visual cue in the form of a button press, previous research has utilized fixation, anti- and pro-saccadic training to examine changes in accuracy to the desired response, which in their study was the execution of an anti-saccade. Despite the differences in training and assessment parameters, together our findings suggest an overall interaction between eye movements and improved accuracy on visual tasks. However, Dyckman and McDowell [27] found a measurable difference in anti-saccade formation after a minimum of three days, whereas our study found significant improvements after only a short training period (18.5 minutes).

A probable neural mechanism for facilitating the observed improvement in response accuracy following eye exercises can be inferred from studies examining trans-saccadic integration, the processing of visual information along the path of an eye movement, which have demonstrated the heightened ability to recognize features of presented objects on a post-saccadic recognition test [38]. The cognitive processes involved with recognizing the shape, color, or other features of an object may also mediate the recognition of target and non-target letters, as was required in our RSVP task. We found very similar results in a previous fMRI investigation of divided attention. Using the same RSVP task employed in this study while subjects performed pro- and anti-saccades, blood-oxygen-level-dependent (BOLD) activity was increased in oculomotor regions of the prefrontal cortex (PFC), including the frontal eye fields and dorsolateral PFC. Additionally, longer saccade latency and more errors were produced in anti-saccade trials with short (200–250 ms) instruction times [35]. That the present investigation showed significant improvements in response accuracy during short and successive presentations of target letters (50 to 133 ms apart) could reflect short-term plasticity and enhancement of neuronal signals in the oculomotor cortical circuit due to a brief training period of eye exercises. Further evidence in support of this putative neural mechanism in mediating increased accuracy for object recognition, such as whole words, comes from a study by Lyle and colleagues [39] that shows enhanced recall of a word following 30 seconds of horizontal eye movements preceded by the study of a word array [39].

Because past research has found pursuit of a moving square through a circle path to stimulate creativity (specifically, originality and flexibility), it comes as no surprise that eye movements may similarly influence cognitive areas [40]. Our study compared improvements in letter identification accuracy between groups. Following eye exercises, a significant improvement in letter identification was found (P<.05, d′ = −0.04 pre-training, d′ = 0.77 post-training). This enhancement in accuracy was not observed in our control group, who performed at a similar level to their pre-training assessment. This is in line with past research, which found horizontal eye movements also improved the recall of laboratory and everyday events [41]. However, this was found solely for horizontal saccade formation, and not for vertical or smooth pursuit saccades. A possible mechanism for this improvement has been further suggested by Murray, Beutter, Eckstein and Stone [42]. On a visual search task, they demonstrated that during saccade formation, one could assess visual objects based on shape. From these findings, we could infer that eye movements increase one’s ability to identify a change in letter shape. Indeed, it was found in our study that participants performed well above chance for observing a change in letter rather than color. If participants were unable to identify the change in letter, they would guess with an accuracy rate of 3.8% (1/26 letters in English alphabet). A study by Brunye, Mahoney, Augustyn and Taylor [43] supports this claim by finding that horizontal eye movements enhance the detection of changes in landmark information, specifically the shapes and locations of objects. However, their findings were only applicable to horizontal eye movements, due to a negligible effect in vertical saccades and fixation, and examined much more complex visual cues. Together these investigations from their respective varying perspectives all lend evidence to increased inter-hemispheric brain activation with the performance of repeated bilateral horizontal eye movements.

Another interesting interaction between object recognition and eye movements comes from research on the interference of neural processing as a result of eye blinks. As demonstrated by Thomas and Irwin [44], voluntary eye blinks interfered with object identification during trans-saccadic partial reports. They named this phenomenon the *cognitive blink suppressioņ* and postulated that this process hinders neural processing of visual stimuli. In relation to our study, a similar suppressive process may exert an influence on letter identification accuracy. However, our study did not involve voluntary blinking, nor did we utilize an eye tracking mechanism to track eye blinks during the experiment. Future studies could incorporate these elements to provide potential clarification on this interaction. Additionally, our future studies will examine the enlarging of pupil dilation, which has now been found to be co-activated during stimulation of a brain structure involved in attention and eye movements in non-human primates [45]. However, we do not feel that this phenomenon is an influential factor in explaining our results because subjects were not required to perform eye movements during the RSVP task.

More recently, vertical saccades have been shown to improve performance on item retrieval, pair association tests, and recall of contextual information, both intrinsic (color) and extrinsic (spatial location) [46]. Further, smooth pursuit studies in preschool children have correlated the inability to follow an instructor’s cue with lower scores on cognitive tests in phonological awareness, path copy and letter recognition [47]. This is to show that these types of eye movements deserve further study to clarify their involvement in enhancing cognition.

Together with our results, we use these lines of research to propose an underlying neural mechanism that facilitates the enhancement of visual attention and memory following eye exercises, even when performed for a very brief training period. Common functional regions of the cortex have been activated during tasks of shifting visual attention and eye movements. This wide-reaching network includes the superior temporal sulcus (STS), intraparietal sulcus (IPS), and portions of the precentral sulcus and medial frontal gyrus [33]. It is important to note that these investigators did not examine changes in accuracy or response time over the course of task performance (i.e., with time, accounting for practice or ceiling effects). As they pertain to our investigation, however, the continued and synchronous activation of this network during the brief eye exercise task may have been sufficient to facilitate short-term plasticity [34]. Similarly, eye exercise may induce neuronal potentiation, effectively “ramping up” preparatory oculomotor signals in dorsolateral prefrontal cortex and frontal eye fields [20], priming these pre-existing neuronal circuits to facilitate the enhancement in response accuracy seen in our results.

We further posit that a similar mechanism can be attributed to the observed improvement in letter identification for the eye exercise group, with previous research showing links between regions related to working memory, attention and eye movements [12]. Although these investigations examine spatial memory, and not declarative memory as in the recall of a presented target letter, they implicate the lateral intraparietal area as an attentional mediator of eye movements, which are executed by the superior colliculus [12]. Training with eye exercise could similarly induce short-term plasticity and/or potentiation of connecting axonal signals in this cortical network, enhancing the availability of neuronal resources in the letter identification portion of the post-training RSVP task. In turn, this priming could facilitate the enhancement in letter identification observed in our results.

The results of our experiment found a performance enhancement for the detection of visual targets when presented in very rapid succession (i.e., separated by one or no distractors) following repetitive eye movements. Although the RSVP task did not demonstrate any differences in reaction time following the eye exercises, there was a significant increase in letter identification accuracy in the experimental group only. Theoretically, these results suggest that a common cortical network that mediates cognition, attention, and oculomotor behavior is capable of undergoing very short-term plasticity, which in turn improves subsequent performance on related tasks. However, more research needs to be done before letter identification through RSVP can be deemed an appropriate tool for measuring the progression of vision therapy, and whether vision therapy is the most effective option for cognitive and eye-related impairments. Further examinations could incorporate eye tracking and a more dispersed regime of visual training to clarify what effects visual therapy may have on the performance of cognitive tasks.
